# Multispecies Probiotic Supplementation in Chronic Kidney Disease (CKD): Insights From Indian Nephrology Experts

**DOI:** 10.7759/cureus.102520

**Published:** 2026-01-28

**Authors:** Pankaj Beniwal, Manish Singla, Deba Prasad Kar, Vinant Bhargava, Naman Chandra, DK Sinha, Vivek Ruhela, Rathan Jha, Rajesh Joseph, Vishnu RS, Nitin Bhosle, Kapil Mehta, Sarita Bajpai

**Affiliations:** 1 Nephrology, Sawai Man Singh (SMS) Medical College, Jaipur, IND; 2 Nephrology, Max Super Speciality Hospital, Mohali, IND; 3 Nephrology, Institute of Medical Sciences (IMS) and Siksha ‘O’ Anusandhan (SOA) Deemed to be University (SMU), Bhubaneshwar, IND; 4 Nephrology, Sir Ganga Ram Hospital, Delhi, IND; 5 Nephrology, Nirmal Hospital, Rishikesh, IND; 6 Nephrology, Heritage Hospital, Varanasi, IND; 7 Nephrology, Shri Mahant Indiresh Hospital, Dehradun, IND; 8 Nephrology, Center for Advanced Research and Excellence (CARE) Hospitals, Hyderabad, IND; 9 Nephrology, Believers Church Medical College and Hospital, Thiruvalla, IND; 10 Nephrology, Sree Uthradom Thirunal (SUT) Hospital, Trivandrum, IND; 11 Nephrology, Kidney Ease Clinic, Mumbai, IND; 12 Medical Affairs, JB Pharma, Mumbai, IND

**Keywords:** chronic kidney disease (ckd), ckd management, gut dysbiosis, probiotics and microbiome, uremic toxins

## Abstract

Chronic kidney disease (CKD) is a progressive condition leading to the accumulation of uremic toxins and systemic complications, eventually resulting in loss of kidney function. One of the major mechanisms behind the CKD complications is gut dysbiosis, a commotion in the gut micro-ecology that aggravates systemic inflammation and worsens the CKD management. Probiotics, through their ability to restore gut micro-ecology, strengthen the intestinal barrier integrity, modulate the immune system, and mitigate systemic inflammation, offer a promising add-on therapy to traditional CKD therapies. Specific probiotic strains, including *Streptococcus thermophilus*, *Lactobacillus acidophilus*, *Bifidobacterium longum*, and *Bacillus coagulans,* have demonstrated antimicrobial effects by promoting commensal bacterial growth and inhibiting pathogenic bacterial growth. Studies indicate that these probiotics reduce uremic toxin levels, such as indoxyl sulfate and p-cresyl sulfate, blood urea nitrogen, urea, and creatinine levels, enhance gut integrity, alleviate inflammation, and are beneficial for patients with CKD stages 3 to 5. While the evidence for probiotic supplementation is promising, further studies are required to establish their long-term benefits, especially in patients with immune deficiency or kidney transplants. Overall, this consensus article provides insights from Indian nephrology experts on the potential benefits of multispecies probiotic supplementation in CKD patients, focusing on improving their quality of life, delaying disease progression, and ultimately augmenting survival outcomes.

## Introduction and background

Chronic kidney disease (CKD) is a major health concern afflicting millions of people globally. Growing prevalence around the globe, especially in India, has drawn the attention of nephrologists and other healthcare professionals, compelling them to focus on different or novel strategies for CKD management [1]. In low- and middle-income countries such as India, late diagnosis and limited access to dialysis and transplantation further increase the clinical burden of CKD, emphasizing the need for supportive and preventive therapeutic strategies.

CKD condition progresses gradually and ultimately worsens over time, resulting in loss of kidney function. CKD is associated with a plethora of systemic complications, including cardiovascular disease, anemia, and bone disorders due to the accumulation of toxins in the bloodstream and eventually in the body [2]. These complications significantly impair quality of life (QoL) and are major contributors to hospitalization and mortality in CKD patients.

Uremic toxins constitute metabolic waste products, such as ammonia/urea and uric acid; gut bacterial metabolites, such as indoxyl sulfate (IS) and p-cresyl sulfate (PCS); and trimethylamine N-oxide (TMAO) [2,3]. Studies indicate that CKD patients exhibit increased production of uremic toxins, such as indole-3-acetic acid (IAA) and trimethylamine N-oxide (TMAO) [3]. Individuals with healthy kidneys eliminate these toxins, including their precursors such as urea, ammonia, and p-cresol; however, patients with CKD demonstrate retention of uremic toxins, and instead of elimination, these are secreted in the gut [4]. The hydrolysis of urea by gut microbes leads to the formation of large amounts of ammonia that raises the luminal pH, resulting in the preferential growth of pathogenic bacteria and a decline in the development of beneficial bacteria [2-6]. This altered composition and disruption of gut micro-ecology, qualitatively as well as quantitatively, is referred to as gut dysbiosis. The consequent increase in ammonia depletes the critical protein that constitutes intestinal epithelial tight junctions, increasing the gut permeability and compromising gut barrier integrity, a phenomenon called “leaky gut” [5,6]. The translocation of bacterial byproducts (e.g., endotoxins), lipopolysaccharides (LPS), uremic toxins, and cytokines from the intestinal lumen into the blood circulation and other bodily compartments triggers systemic inflammation and increases oxidative stress [7]. This perpetuating systemic inflammation is believed to contribute to cardiovascular and other CKD-related complications.

Moreover, patients with CKD are recommended to follow dietary regimens that may involve controlled protein intake and variable fiber consumption, depending on disease stage and metabolic status, which, in many patients, may reduce intake of fibers, proteins, and carbohydrates. A lack of fermentable fibers decreases the growth of saccharolytic gut bacteria, such as *Bifidobacteria* and *Lactobacilli*. These beneficial gut bacteria are known to produce short-chain fatty acids (SCFAs) such as acetate, propionate, and butyrate that preserve gut health. Low production of these SCFAs has been associated with leaky gut and worsening of CKD condition [1,4]. In addition, CKD patients are often on multiple medications, including antibiotics, which alter the relative bacterial count, diminishing the microbiome diversity, while phosphate binders and ion exchange resins might affect intestinal microbial growth; however, they result in decreased colonic transit [8].

Thus, the role of the gut-kidney axis and the interplay of gut dysbiosis in inducing systemic inflammation and immune modulation in CKD patients has been gaining considerable interest. Gut-targeted therapeutic strategies aiming to restore gut health have been a central point of research in CKD patients, one of which is probiotic supplementation [4]. Probiotics are live microbes, typically beneficial bacteria, that soothe gut health, restore the imbalanced micro-ecology, and provide potential health benefits when taken in adequate amounts. Multispecies probiotic formulations have been investigated in CKD for their potential to influence gut dysbiosis, uremic toxin generation, and inflammatory pathways, although clinical results remain heterogeneous. Primarily, the multiple strains aid in re-establishing the gut microflora by favoring the growth of beneficial bacteria, such as *Lactobacillus* and *Bifidobacterium*, and suppressing the growth of pathogenic bacteria, alleviating the vicious effects of gut dysbiosis [9]. Secondly, probiotics enhance the integrity of the gut barrier, which may reduce gut permeability and limit the translocation of endotoxins and pathogenic bacteria across the intestinal wall. Thirdly, studies indicate that specific probiotic strains reduce uremic toxins in the bloodstream by metabolizing them in the gut prior to their entry into systemic circulation. Lastly, a probiotic-induced series of events might mitigate systemic inflammation, modulate the immune system, and reduce oxidative stress by augmenting anti-inflammatory cytokines and diminishing pro-inflammatory cytokines, resulting in a sustained systemic environment. Moreover, probiotics induce antibacterial effects by reducing the local pH in the intestinal lumen, competing with opportunistic bacteria for nutrients, and eventually eliminating them from the system [10,11]. Although these mechanisms are derived largely from microbiome and gastrointestinal research and may not apply uniformly across all probiotic formulations or CKD populations.

Modulation of the gut dysbiosis, immune system, and systemic inflammation with the supplementation of multispecies probiotics has proven efficacy from more than a decade in CKD patients, with multiple small randomized and observational studies reporting favorable changes in surrogate markers such as uremic toxins, inflammatory mediators, gut permeability, and patient-reported outcomes. However, the available evidence remains limited by sample sizes, heterogeneity of probiotic formulations, and variability in study design. This article provides an extensive discussion on managing CKD patients with multispecies probiotic supplementation, evaluating the beneficial role of multispecies probiotics in modulating factors that contribute to CKD progression through expert panel discussions.

## Review

Aim of consensus

This consensus paper aims to provide a comprehensive understanding of the role of a multispecies probiotic formulation in managing CKD. This consensus focuses on two key objectives. The foremost objective is to elucidate the therapeutic potential of multispecies probiotics in addressing the gut dysbiosis commonly associated with CKD. By establishing a clearer understanding of how probiotics can help restore the gut microbiota, strengthen the intestinal barrier, and reduce inflammation, this consensus aims to provide nephrologists with evidence-based insights into how probiotics can be integrated into CKD management. This consensus focuses on a formulation comprising four probiotic strains: *Streptococcus thermophilus*, *Lactobacillus acidophilus*, *Bifidobacterium longum*, and *Bacillus coagulans*. This multi-strain formulation was chosen because every strain individually demonstrated the ability to modulate the gut environment, improve gut integrity and host-immune system, and provide anti-inflammatory effects, essential to CKD management. For instance, *Streptococcus thermophilus* reduces blood urea nitrogen (BUN), uric acid, and p-cresol in plasma and increases quality of life (QOL) [12-15].* Lactobacillus acidophilus *reduces the concentration of uremic toxins in the bloodstream and averts pathogenic bacteria growth in the small intestine. *Bifidobacterium longum* removes putrefactive bacteria, phenolic, and indole toxic metabolites, decelerating the progression of CKD [16,17]. *Bacillus coagulans* alters intestinal permeability, preventing the translocation of toxins. These individual probiotic strains, when formulated together, could be targeted at alleviating the systemic effects of CKD, establishing a central part of a multifaceted therapeutic strategy.

Methodology

A total of ten digital expert discussions were convoked from April 2024 to March 2025 to collect insights from Indian nephrology experts regarding the role of multispecies probiotic supplementation in CKD. These discussions included 60 Indian nephrologists with a wide range of clinical expertise and knowledge in CKD management. The main purpose of these meetings was to provide insights into (i) the role of multispecies probiotics in tackling gut dysbiosis in CKD patients; (ii) assess the beneficial effect of probiotics on the several critical factors including systemic complications of this multifaceted CKD condition; and (iii) identification of subcategories of CKD patients who are deemed fit to be recommended for multispecies probiotic supplementation and those for whom caution should be warranted. 

A comprehensive literature review was performed using the PubMed database to identify relevant articles published between 2003 and 2024. The search was conducted using specific keywords such as “Chronic Kidney Disease,” “Probiotic supplementation,” “Gut dysbiosis,” “Gut dysbiosis and chronic kidney disease,” “multispecies probiotics and CKD,” and “Uremic toxins and probiotics.” Key articles and evidence were shortlisted based on their relevance to the role of probiotics in CKD management. These articles were circulated among the expert panel members as pre-reading material to facilitate informed discussions during the advisory board meetings. In each advisory board meeting, a qualitative question-and-answer (Q&A) format was utilized to promote interactive discussions among nephrologists. Post-expert discussion, the key expert opinions were noted and assimilated to produce consensus insights, which are summarized in this manuscript.

Key areas of discussion

Probiotic Effects on CKD

Improving intestinal barrier integrity: The expert group discussions highlighted the potential of multispecies probiotics to improve the integrity of the intestinal barrier in patients with CKD. Experts emphasized that the impairment of the gut barrier in CKD plays a crucial role in the development of endotoxemia and systemic inflammation, which in turn may expedites the progression of CKD. A meta-analysis of eight studies including 261 patients, published by Jia et al., concluded that supplementation of probiotics might decrease the levels of p-cresyl sulfate (PCS) and elevate IL-6 levels in CKD patients known to enhance the tight junction proteins and proliferate intestinal epithelial cells to improve the gut permeability [18]. In a similar line of context, a study administering a synbiotic (probiotic (including *Lactobacillus casei* and *Bifidobacterium animalis*) + prebiotic) formulation to CKD patients reported decreased permeability of the small intestine as a result of lower levels of indoxyl sulfate (IS) levels, an indicator for intestinal permeability [19]. Experts also discussed and opined that the enhanced levels of SCFA-producing bacteria are biologically associated with improved gut integrity. A study conducted by Huang and his colleagues indicated that a *Lactobacillus* mix containing *Lactobacillus paracasei *and* Lactobacillus plantarum* aids in restoring commensal bacteria and elevating the abundance of SCFA producers, which are essential for the tightening of the intestinal lining that eventually decreases systemic inflammation. These SCFAs also fuel intestinal epithelial cells and increase mucin secretion, protecting gastrointestinal mucosa from bacterial insults [20]. Further, Araujo et al. demonstrated that probiotics comprising *Lactobacillus plantarum*, *Lactobacillus rhamnosus*, *Bifidobacterium bifidum,* and *Bifidobacterium longum *strains significantly strengthen the gut barrier integrity by downregulating the expression of syndecan-1 in the serum samples of CKD patients on hemodialysis (HD). Decreased serum levels of syndecan-1, a well-established indicator known for maintaining the tight junctions of the intestinal barrier, suggest restricted translocation of endotoxins and other toxins that otherwise aggravate the CKD condition [21]. Hence, by lowering the levels of IS and PCS along with the increase in the SCFA levels, thereby enhancing the tight junction proteins, these probiotics stabilize the barrier function and reduce the permeability.

Anti-inflammatory and antimicrobial effects: Building on findings from several randomized clinical trials and real-world evidence, experts highlighted the anti-inflammatory and antimicrobial potential of multispecies probiotics in patients with CKD. Wang et al. and his colleagues found that the probiotic containing *Bifobacterium bifidum*, *Bifidobacterium catenulatum*, *Bifidobacterium longum*, and *Lactobacillus plantarum* down-regulated the levels of pro-inflammatory cytokines, such as tumor necrosis factor-α (TNF-α), IL-5, IL-6, and endotoxin, while up-regulated the levels of anti-inflammatory IL-10 in the serum samples of CKD patients on peritoneal dialysis (PD) post after six months of treatment [22]. This is to be noted that these findings are supportive and hypothesis-generating, not proof of clinically meaningful anti-inflammatory benefit. Probiotics containing *Bifidobacterium bifidum *(BGN4) and *Bifidobacterium longum* (BORI) showed significant upregulation of anti-inflammatory monocytes CD4+ CD25+, regulatory T cells (Tregs) from 3.5% to 8.6%, (p < 0.05) and significant downregulation of the pro-inflammatory monocytes; CD14+ CD16+ from 310/mm^2^ to 194/mm^2^, (p < 0.05) post six months' intervention in HD patients [23]. Natarajan et al. in 2014 conducted a double-blind clinical trial of a probiotic formulation (containing 30 billion colony-forming units (CFU) of *S. thermophilus* KB 19, *L. acidophilus* KB 27, and *B. longum* KB 31) in CKD patients on dialysis, indicating reduced trends in C-reactive protein (CRP) and total indoxyl glucuronide, emphasizing on its anti-inflammatory potential [24]. A recent study also highlighted the anti-inflammatory potential of probiotics, demonstrating that their administration dropped CRP levels in patients with mild CKD [25], although clinical outcome benefits were not assessed in these trials. Additionally, probiotics illustrated an antimicrobial effect by promoting the growth of commensal bacteria, which inhibit the growth of exogenous and endogenous harmful bacteria [14,20,25,26]. Probiotics showed an increase in the SCFA-producing bacteria, which may inhibit the growth of the pathogenic bacteria by lowering the luminal pH and secreting antimicrobial proteins [20]. However, microbiome modulation is complex, and not all microbial shifts are necessarily beneficial, particularly in immunocompromised or advanced CKD patients. The experts of the panel collectively suggest that probiotics may alleviate inflammation in CKD by elevating anti-inflammatory markers and increasing saccharolytic bacteria to inhibit endotoxemia potentially reducing CKD-related life-threatening complications. However, these biological effects should not be interpreted as proof of reduced hospitalization, infection risk, or disease progression in the absence of outcome-driven trials.

Competition with pathogenic bacteria: The ability of probiotics to compete with harmful and morbific bacteria for nutrition and adhesion to the intestinal lining or lumen was also identified as a potential benefit in CKD management. Experts documented that intestinal dysbiosis in CKD promotes the flourishing of pathogenic bacteria, which generate uremic toxins that promote functional loss of the kidney. Three independent groups of researchers, Cruz-Mora et al., Simeoni et al., and Rossi et al., showed that the supplementation of multispecies probiotics results in increased bacterial growth of beneficial bacteria, i.e., *Lactobacillales* and *Bifidobacteria*, and decreased the growth of CKD patients after the short-term intervention in patients with CKD and end-stage renal disease (ESRD). These beneficial bacteria outcompete the pathogenic microorganisms belonging to different genera by colonizing the gut abundantly [14,20,25,26]. These studies suggest that dysbiosis, present in CKD can be effectively corrected by the administration of high-quality probiotics, with improvement of inflammatory indices (PCS), iron status, and intact parathyroid hormone (iPTH) stabilization. This competitive exclusion might aid in reducing the burden of pathogenic bacteria on the gut, regulating the production of toxins like indoxyl sulfate and p-cresyl sulfate, which cause the systemic effects of CKD [10,11].

Clinical Evidence of Probiotics on Uremic Toxins and Uremia

The experts in the panel observed and noted the clinical benefits of probiotic supplementation in CKD patients, including reductions in uremic toxins, such as IS, PCS, BUN, urea, and creatinine levels. Several clinical trials and real-world evidence reported improvements in these kidney function markers. Additionally, experts discussed data on probiotics facilitating the stabilization of the glomerular filtration rate (GFR), a crucial marker for kidney function. An earlier study indicated that a probiotic containing *Bifidobacterium longum* decreases the serum levels of IS post five weeks of its oral ingestion by CKD patients on HD [15]. Oral ingestion of *L. casei *Shirota and *B. breve* strain for four weeks in HD patients demonstrated a decrease in the PCS levels, a marker of uremic toxins [27]. Guida et al. also demonstrated a similar trend of reduced PCS levels with probiotic administration in patients with CKD stages 3 and 4 [28]. In a similar context, Ranganathan and his team conducted a series of randomized clinical trials and analyzed the effect of probiotics containing multiple strains on the uremic toxin in patients with CKD stages 3-5. Post six months of trials, they found a significant reduction in the levels of BUN and a considerable, however non-significant reduction in creatinine and uric acid in the enrolled subjects [12,13]. A significant reduction in the blood urea level was observed by the administration of *L. casie *Shirota in patients with CKD stages 3 and 4 treated with the 16 x 10^9^ dose for eight weeks [29]. A study published by Kalidindi et al. suggested that multispecies probiotics containing *S. thermophilus*,* L. acidophilus*, *B. longum*, and *B. coagulans* result in a significant reduction in BUN, serum levels of creatinine and uric acid, and improvement in the estimated GFR with an increase of >35% [16]. A study with probiotics containing a strain of *S. thermophiles*, *L. acidophilus*, and *B. longum*, in combination with prebiotics and a low-protein diet significantly reduces the declining rate in GFR, after six months of the intervention in patients with CKD stages 3-5 [30]. Seventeen non-dialysis patients, those who received probiotics containing *L. acidophilus*, *L. casei*, and *B. lactis*, vs those who received a placebo for 12 weeks, demonstrated a reduction in the serum level of IS (ΔIS -21.5% vs. 5.3%, P<0.001) and improved eGFR (ΔeGFR 12% vs. 8%, P=0.029) [31]. The clinical data available over the years consistently highlight the probiotics' ability to reduce BUN, stabilize or improve GFR, and modulate uremic toxins such as IS and PCS. However, results across these studies have been heterogeneous [32]. The effects on serum creatinine delivered variable outcomes, probably due to variances in study designs, probiotic strains, and treatment durations. The list of clinical studies reporting the usage of probiotics in CKD is provided in Table 1.

**Table 1:** Clinical studies reporting the use of probiotics in CKD. BUN: blood urea nitrogen, QOL: quality of life, PCS: p-cresyl sulfate, IS: indoxyl sulfate, GFR: glomerular filtration rate, CKD: chronic kidney disease.

Author (year)	Stage of CKD	Probiotics	Duration	Dialysis status	Inferences
Ranganathan et al. (2009) [12]	Stage 3-4	*Lactobacillus acidophilus*, *Bifidobacterium longum*, *Streptococcus thermophilus*	Six months	Non-dialysis	Reduction in uremic toxins with reduced BUN, uric acid levels, improved quality of life
Ranganathan et al. (2010) [13]	Stage 3-4	*Lactobacillus acidophilus*, *Bifidobacterium longum*, *Streptococcus thermophilus*	Six months	Non-dialysis	Reduction in uremic toxins with decreased BUN and improved well-being
Nakabayashi et al. (2010) [27]	ESRD	*Lactobacillus casei*, *Bifidobacterium breve*	Four weeks	Hemodialysis	Reduction in uremic toxins with significant decrease in serum p-cresol and improvement in GI symptoms
Cruz-Mora et al. (2014) [14]	ESRD	*Bifidobacterium lactis*, *Lactobacillus acidophilus*, Inulin (prebiotic)	Two months	Hemodialysis	Enrichment of commensal bacteria with increased *Bifidobacterium *counts resulting in improved gut microbiota and reduced GI symptoms
Guida et al. (2014) [28]	Stage 3-4	*Lactobacillus casei*, *Bifidobacterium lactis*	Four weeks	Non-dialysis	Reduction in uremic toxins with lowered plasma p-cresol levels
Natarajan et al. (2014) [24]	ESRD (dialysis patients)	*Lactobacillus acidophilus*, *Bifidobacterium longum*, *Streptococcus thermophilus*	Two months	Dialysis	Moderate anti-inflammatory effects with stable QOL
Miranda Alatriste et al. (2015) [29]	Stage 3-4	*Lactobacillus casei *Shirota	Eight weeks	Non-dialysis	Reduction in uremic toxins with the reduction of blood urea >10% at higher dose
Wang et al. (2015) [22]	-	*Bifidobacterium bifidum*, *Bifidobacterium catenulatum*, *Bifidobacterium longum*, *Lactobacillus plantarum*	Six months	Peritoneal dialysis	Anti-inflammatory effect with the reduction in endotoxin, tumor necrosis factor-α (TNF-α), IL-6 levels resulting in preserved renal function
Firouzi et al. (2015) [33]	Early-stage CKD	*Lactobacillus plantarum*, *Bifidobacterium lactis*	12 weeks	Non-dialysis	Reduction in the urea levels in type 2 diabetes patients with early-stage CKD patients having high urea levels
Rossi et al. (2016) [26]	Stage 4 or 5	*Lactobacillus acidophilus*, *Bifidobacterium bifidum*, Inulin (prebiotic)	Six weeks	Non-dialysis	Reduction in uremic toxins, such as PCS and IS, resulting in improved gut microbiome
Pavan et al. (2016) [30]	Stage III-V	Combination of prebiotic fiber and *Lactobacillus *or *Bifidobacterium *strains	12 months	Non-dialysis	Stabilization of eGFR levels with lower GFR decline in the probiotic group resulting in delayed CKD progression
Simeoni et al. (2018) [25]	Stage 3a	*Lactobacillus acidophilus*, *Bifidobacterium longum*, with dietary fibers	Varied (open-label study)	Non-dialysis	Improvement in gut dysbiosis with increased beneficial gut bacteria, reduced indican, and CRP levels
Cosola et al. (2021) [19]	Stage 3b-4	*Bifidobacterium lactis*, *Lactobacillus acidophilus*, with prebiotic fiber	Two months	Non-dialysis	Reduction in uremic toxins, such as IS, resulting in improved gut symptoms and reduced intestinal permeability
Mitrovic et al. (2022) [31]	Stage 3-4,	*Lactobacillus acidophilus*, *Lactobacillus casei*, *Bifidobacterium lactis*, Inulin (prebiotic)	12 weeks	Non-dialysis	Reduced uremic toxins, such as IS and p-cresol, with stable renal function indicated by improved eGFR, decreased CRP levels
Choi et al. (2022) [23]	Maintenance HD patients	*Bifidobacterium bifidum *(BGN4), *Bifidobacterium longum *(BORI)	Three months	Hemodialysis	Anti-inflammatory effect with decreased calprotectin, IL-6, inflammatory monocytes, and increased pro-inflammatory marker Tregs
Sharma et al. (2022) [17]	Stage 3-4	Multi-strain probiotics, including *Lactobacillus *and *Bifidobacterium *species	180 days	Non-dialysis	Anti-uremic and anti-inflammatory effect via a significant drop in blood urea and CRP levels
Araujo et al. (2023) [21]	ESRD	*Lactobacillus plantarum*, *Lactobacillus rhamnosus*, *Bifidobacterium bifidum*, *Bifidobacterium longum*	Three months	Hemodialysis	Improvement in the intestinal barrier permeability with the reduced syndecan-1 level
Kalidindi et al. (2024) [16]	Stage 3-4	*Lactobacillus acidophilus*, *Bifidobacterium longum*, *Streptococcus thermophilus*	Six months	Non-dialysis	Reduction in uremic toxins, such as IS, PCS, BUN, and creatinine levels, resulting in improved QOL

Consensus findings

Stage-Specific Benefits

CKD stages are calculated by eGFR levels, which reflect kidney function. Lower eGFR means decreased kidney function and impending progression to kidney failure. An eGFR >90 mL/min/1.73 m² is normal, while <90 marks the CKD onset, progressing from stages 1 through 5: Stage 1: eGFR ≥ 90, with evidence of kidney damage. Stage 2: eGFR 89-60, mild kidney function loss. Stage 3a/3b: eGFR 59-45/44-30, a moderate decline in kidney function. Stage 4: eGFR 29-15, a severe decline in kidney function. Stage 5 (ESRD): eGFR <15, indicating kidney failure, requiring dialysis or transplant [32]. During the expert discussions, the panelists concurred that probiotic supplementation offers stage-specific benefits in patients with CKD. The experts opined and highlighted that the beneficial effects of the multispecies probiotic formulation are more prominent in patients with CKD stages 3 to 5 [12,13,16,17,27,29,31]. Studies indicate that the kidney functions in these advanced stages of CKD are significantly impaired, resulting in the accumulation of higher levels of uremic toxins in the bloodstream. The experts noted that the majority of studies enrolling the patients with CKD stages 3-5 showed that probiotics formulations abridged the burden of these toxins by modulating gut microflora and augmenting the breakdown of toxic metabolites such as urea, IS, and PCS. This is particularly appropriate in advanced stages of CKD, where the gut-kidney axis is compromised. The panelists agreed and emphasized that the administration of multispecies probiotics shows promising effects in all CKD patients, with the strongest biological and clinical signals observed in stages 3-5, when the kidneys are most damaged, and the systemic insult of gut dysbiosis is most severe. 

Impact on Symptoms

The expert panel also formed a consensus on the noticeable enhancements seen in patients with CKD-related symptoms post probiotic supplementation. These observations reflect both findings from small clinical studies and real-world clinical experience reported by nephrologists. Several nephrologists testified, noting improvements in patients with augmented urea levels, a symptom of CKD that causes fatigue, loss of appetite, and nausea. By decreasing the levels of BUN, urea, and other uremic toxins and restoring the gut microbiota in CKD patient’s studies indicated improvement in the CKD-related uremic and gastrointestinal symptoms. The efficacy of multi-strain bacterial cells for 12 weeks in type 2 diabetic patients revealed that probiotics decrease urea levels, especially in overweight and obese individuals [33]. Notably, these findings suggest metabolic and uremic modulation rather than direct effects on body weight. In addition, panel members highlighted improvements in patients struggling with obesity, a common comorbidity in CKD that exacerbates the disease's progression. By promoting an improved gut microbiota and decreasing systemic inflammation, probiotics were reported to help some patients accomplish modest weight loss, as well as expansions in metabolic indicators, which may contribute to better overall outcomes in CKD.

Ideal Dosage

Ideal dosage of the probiotic formulation for CKD patients was another key point of discussion among the nephrologists. The experts critically reviewed the outcomes of clinical studies and real-world evidence and highlighted that the dosage of multispecies probiotics ranged widely from 16 × 10^9^ CFU to 2.0 × 10^12^ CFU. They emphasized and agreed that the studies using ≥15 billion CFU of probiotics containing multiple strains showed the most beneficial effect in CKD patients. Ranganthan et al. established the safety of multispecies probiotics with the usage of doses of up to 180 billion CFU/day for two months and showed improved uremic symptoms with using 90 billion CFU/day of probiotics for six months in CKD patients [12,13]. A study comparing the efficacy of Lobun Forte and Renadyl containing multiple strains by supplementing CKD patients with 45 billion CFU/capsule, twice daily for six months. With this dosage, the probiotics showed beneficial effects in improving multiple factors that contribute to the CKD condition, including increased gut integrity, restoration of gut microbiota, elevated levels of anti-inflammatory markers, reduced uremic symptoms, and stabilization of eGFR levels [16]. Another study conducted by Sharma et al., corroborated the above-mentioned finding by using 45 billion CFU/capsule, bis in die (twice daily) (BID) for six months leading to the reduction in the blood urea and pro-inflammatory CRP levels, thereby, slowing the progression of CKD [17]. After reviewing clinical data and expert insights, the panel concluded that doses in the range of approximately 30-90 billion CFU per day, including regimens such as 45 billion CFU twice daily, have been most commonly studied and appear biologically active in CKD and that this dose is sufficient to achieve the desired beneficial effects. This dosage was found to be efficacious in improving the gut health of CKD patients without causing any deleterious side effects. Experts agreed that this duration and dosage are appropriate to diminish systemic inflammation, balance gut microbiota, and decrease urea levels in CKD patients. Expert also emphasized on the fact that long-term safety in broader CKD populations remains insufficiently characterized, and that cautious use and monitoring are warranted.

Non-dialysis Patients

The nephrologists also precisely discussed the benefits of probiotic supplementation in non-dialysis CKD patients. Many studies were conducted in patients with CKD stages 3 and 4, where dialysis is not required. The supplementation of probiotics tapped into the gut-kidney axis of the patients showed improvements in gastrointestinal symptoms, such as constipation, thereby promoting weight gain [12,13,16,17,27,29,31]. These studies primarily evaluated short-term symptom and biomarker changes rather than long-term renal outcomes. The experts documented that probiotics via balancing the gut microbiota and reducing uremic toxins can stimulate appetite and encourage the absorption of nutrients leading to weight gain for those who struggle with eating. Such effects may contribute to improved nutritional balance in selected individuals, rather than uniform weight gain across all patients. The improvements in the uremic and gastrointestinal symptoms improve quality of life and potentially impede the need for dialysis by alleviating the patient's condition through gut-mediated probiotic therapy.

Quality of Life Improvement

The use of probiotics in CKD management has shown promising benefits in enhancing patients' quality of life, with many reporting improved overall well-being and motivation to continue usage even in advanced stages. Clinical observations suggest that probiotics help alleviate symptoms such as anorexia and nausea while improving appetite and managing constipation, contributing to better nutritional intake and overall health. Additionally, experts have opined that probiotics have a positive impact on delaying CKD progression, further reinforcing their role as a supportive therapy that enhances both physiological and psychological well-being in CKD patients. However, no probiotic trials in CKD have demonstrated definitive improvements in validated quality-of-life scores or mental-health outcomes, and any such effects should be considered exploratory.

Cautionary points

The experts pointed out that probiotics supplementation is safe with minimal side effects for the broader population. However, they raised their concern and discussed the potential contraindications for probiotic use, especially in groups of individuals who possess compromised immunity and patients who have undergone kidney transplantation. CKD patients with advanced stages or ESRD possess weakened immune systems, and the administration of live microorganisms in these patients could bestow potential risks. Immunocompromised individuals with advanced-stage CKD might catch infections from the abundance of even beneficial bacteria, including the commonly used *Lactobacillus* and *Bifidobacterium* in the probiotics, leading to sepsis or other complications [34]. Therefore, the experts noted this probability and suggested that probiotics should be recommended with caution in these patients and they should be carefully monitored for any adverse effects.

Experts also warranted caution for patients who undergo kidney transplants and are on immunosuppressive therapy. In these patients, immunosuppressants reduce the body’s ability to fight against any infections; supplementation with live probiotics might trigger unintended complications in the body, which could become lethal for the patients. Although few studies concluded the beneficial effect of probiotics in patients after kidney transplantation, caution is advised for such patients [35]. It has to be noted that this is a precautionary expert opinion rather than a guideline-based prohibition. The experts opined and formed a consensus that, in such cases, physicians should implement great care and possibly prevent probiotic supplementation completely unless there is a convincing indication of benefit. Additionally, experts emphasized that individual risk assessments are critical before the usage of probiotics in these groups.

Limitations

Despite the available large body of evidence for the promising potential of probiotic supplementation in CKD, the expert panel emphasized the requirement for larger clinical trials with longer follow-ups to further validate the findings of small cohorts. The current body of evidence is limited in its designing and sample size; therefore, well-designed, randomized controlled trials enrolling diverse patient populations, including non-dialysis and dialysis patients, are essential to deliver more vigorous data on the long-term effects of probiotics on kidney function, inflammation markers, and patient survival rates. Moreover, studies exploring the interactions of probiotics and standard CKD therapies should be conducted to understand the synergy that can postulate different therapeutic strategies.

## Conclusions

Based on the available data, the experts formed a consensus recommending the usage of multispecies probiotics as supportive therapy rather than a standalone therapy for CKD management. Probiotic supplementation has shown promising biological and symptomatic signals in patients with CKD stages 3-5, targeting multiple factors contributing to the multifaceted CKD condition. The potential of probiotics to improve the gut barrier function with enhanced intestinal integrity and decreased permeability, to exert anti-inflammatory effects, and to compete with opportunistic bacteria makes them a valuable addition to the holistic management of CKD. The panel noted that these effects may contribute to improvements in gastrointestinal symptoms and uremic toxin burden, which could support patient well-being. The panelists also agreed that clinicians should prescribe probiotic supplements into the broader treatment regimen for patients with advanced-stage CKD while carefully monitoring the immune-deficient and post-transplant patients, keeping potential contraindications in mind.

Although larger clinical trials with longer follow-ups are required to better understand the clinical outcomes and potential survival benefits of probiotics, the current evidence and future studies can strengthen the possibility of making probiotics a crucial part of CKD management. This approach could aim at improving the quality of life, decelerating disease progression, and ultimately augmenting survival outcomes for patients with CKD. Future research should focus on multicenter randomized controlled trials with hard clinical endpoints, standardized quality-of-life measures, microbiome-based stratification, safety monitoring, and comparative evaluation of probiotic formulations to determine whether these biological effects translate into meaningful long-term clinical benefit.
